# Getting Through the Crisis Together: Do Friendships Contribute to University Students’ Resilience During the COVID-19 Pandemic?

**DOI:** 10.3389/fpsyg.2022.880646

**Published:** 2022-05-16

**Authors:** Vanessa Kulcar, Tabea Bork-Hüffer, Ann-Malin Schneider

**Affiliations:** ^1^Department of Psychology, University of Innsbruck, Innsbruck, Austria; ^2^Disaster Competence Network Austria, Vienna, Austria; ^3^Department of Geography, University of Innsbruck, Innsbruck, Austria; ^4^Independent Researcher, Innsbruck, Austria

**Keywords:** COVID-19, relationships, social networks, social resource, resilience, young adults, emerging adulthood, wellbeing

## Abstract

Social contacts and social support represent resources that contribute to resilience. However, the COVID-19 pandemic and the associated measures, including contact restrictions, posed challenges for young adults’ social networks, in particular for their friendships. Employing a mixed-method approach, we investigated the pandemic’s effects on friendships and their role in successfully navigating the crisis. We combined a qualitative approach based on narratives and in-depth interviews and a quantitative approach based on online surveys focusing on university students in Austria. Longitudinal data collections allowed investigating changes and developments as the pandemic progressed. Results indicate profound challenges for participants’ friendships and difficulties in both building new and maintaining existing friendships. This also impaired the provision of social support by friends, scattering participants’ social resources and diminishing their resilience rather than strengthening it. Altogether, the results of this longitudinal study suggest a lasting negative effect of the pandemic on friendships for students.

## Introduction

COVID-19 emerged in 2019, spread throughout the world, and was declared a pandemic in March 2020 (World Health Organization, 2020). The pandemic persists over 2 years later and caused profound changes in the lives of people across all countries, population groups, and ages. However, early on, research emerged that observed young adults, especially students, as particularly vulnerable to the mental health impacts of the crisis (Braun et al., 2020; Salari et al., 2020; Xiong et al., 2020). Emerging or young adults are in a stage in their lives that is characterized by instability and insecurity (Arnett, 2000), for example regarding their education, their career paths, and their social networks, which leaves them vulnerable to the increased insecurities due to the crisis (Alonzi et al., 2020). Specifically, university students often move away from their families to begin their studies which deprives them of their established social networks to rely on for support during stressful times. At the same time, distance learning impedes establishing new contacts and making friends among fellow students (Besser et al., 2020). Understanding how friendships are affected can be crucial for understanding the mental health impacts of the COVID-19 pandemic and the associated restrictions as differences in friendships and social support by peers might be a predictor of how students cope with the crisis and who is resilient.

In previous health crises, a substantial part of the affected population has been shown to be resilient (Bonanno et al., 2008). There are different definitions of resilience. In general, individuals who do not experience significant mental health or functional impairment during stressful and straining events can be considered resilient (Southwick et al., 2014). Resilience thereby depends on the availability of resources that help cope with the event (Abramson et al., 2015; Hobfoll et al., 2015). Abramson et al. (2015) defined social capital as one of four resilience attributes that enable resilience. On the level of individuals, social capital includes family, friends, and other social contacts as well as perceived social support. They further assumed that social support can activate resilience. For instance, through social contacts, knowledge and material assistance can be provided and emotion regulation can be supported which assists the individual in adopting adaptive coping strategies (Abramson et al., 2015). Through this mechanism, better outcomes regarding mental and physical health can result. This is what Cohen and Wills (1985) termed the buffer model of social support whereby social support buffers the negative effects of stressful events or circumstances. But social contacts are not only helpful during stressful events but also exert general beneficial effects on wellbeing (Cohen and Wills, 1985). Baumeister and Leary (1995) assumed social connection and belonging to be a fundamental human need. If this need is not met, negative effects up to psychopathological symptoms and disease can be the result. When individuals have enough social contact, they can trust that support is accessible when needed (Barrera, 1986). Therefore, the availability of social support can be assumed to contribute to resilience while a lack of social contacts has to be considered a vulnerability factor.

The COVID-19 crisis can be considered a stressful and straining event for most people. Therefore, social contacts and support might be particularly important during this time. Meanwhile, the pandemic and associated measures, including contact restrictions, are associated with increases in loneliness and social isolation. Studies comparing loneliness before and during COVID-19 found increased loneliness since the start of the pandemic compared to before, both in the general population (Bu et al., 2020) and in students (Lee et al., 2020). Generally, student status was reported to be an amplified risk factor for loneliness during the pandemic (Bu et al., 2020). In a study among university students in the United States at the beginning of the pandemic, 86% reported feeling socially isolated (Son et al., 2020) and in young German adults, 42% disclosed that they felt lonelier compared to before the pandemic (Lippke et al., 2021).

This vulnerability of young adults to feelings of loneliness and isolation can be attributed to the transition stage into adulthood that enhances their need for social contact. Young people need to meet various tasks growing up including the establishment of a successful social life and meaningful relationships (Roisman et al., 2004; Neyer and Lenhart 2007). During the transition into adulthood, attachment functions are transferred from family to peers (Hazan and Zeifman, 1994; Fraley and Davis, 1997). Research has demonstrated that the need for contact and proximity is typically met by peers and peers are most important to young adults for providing comfort and emotional support (Hazan and Zeifman, 1994; Fraley and Davis, 1997). Therefore, friends are deemed critical to this transition period which is also reflected in survey results among young Dutch adults during the COVID-19 pandemic among whom 94% indicated that friends are their source of social support (van den Berg et al., 2021). Simultaneously, due to the measures, social contacts were often restricted to the household as a core living unit. Long et al. (2021) hypothesized that loose contacts or newly established friendships might therefore be easily lost. This particularly applies to university students as they often establish new networks and make new friends during their studies. These new ties might not be strong enough to withstand the strains during the contact restrictions. Therefore, contacts with peers suffered more than contacts with family (Andresen et al., 2020; Wu et al., 2021; Zhang et al., 2021). This effect might be amplified as friendship relations appear to be less stable and require more maintenance including frequent contact and joint activities compared to family relations (Roberts and Dunbar, 2011). Further, spontaneous and unplanned interactions usually provide opportunities for both establishing and maintaining friendships as well as for low-threshold support (Long et al., 2021). These opportunities are largely lost due to the contact restrictions. Receiving support might therefore be bound to deliberately contacting people and asking for it, which constitutes a higher threshold. Correspondingly, MBA students in the United States reported an increased need for social support but friendship ties were not maintained between the students as their university switched to online teaching (Jo et al., 2021). This was partly due to the difficulties of having to arrange specific online appointments, technical issues, and problems of receiving emotional support online.

These difficulties in establishing and maintaining contacts as well as in receiving support from relationships have implications for wellbeing and mental health. Generally, loneliness is associated with mental health impairments during this pandemic (Liu et al., 2020; Xiong et al., 2020; Mäkiniemi et al., 2021; Rosenberg et al., 2021) while more social interactions were associated with better mental health outcomes (Forbes et al., 2021). More specifically, social support is associated with better mental health outcomes (Szkody et al., 2021; Turska and Stepien-Lampa, 2021; Wu et al., 2021). However, results on the social support provided by friends and its association with mental health are ambiguous. On one hand, there are studies reporting beneficial effects of contact with friends as well as friend support. Adults experienced more positive affect after they had had contact with their friends in a study in Ireland (Lades et al., 2020) and university students in Hong Kong reported fewer depressive symptoms when they received more peer support (Sun et al., 2020). Additionally, adults in the United States experienced more posttraumatic growth with higher support from their friends (Northfield and Johnston, 2021). On the other hand, there are studies reporting no effects of friend support. In young adults in the United States (Liu et al., 2020) and in the Netherlands (van den Berg et al., 2021), friend support was not associated with better mental health. Liu et al. (2020) assumed that peers might not be able to adequately support each other during this crisis as they are all confronted with similar problems and cannot provide guidance or a different perspective. Contrarily, Nitschke et al. (2021) argued that social contacts might be especially helpful during the COVID-19 crisis as everyone experienced similar situations and therefore there is a high level of understanding.

Simultaneously, the COVID-19 crisis does not necessarily have negative impacts on relationships and the provision of social support as social networks are often able to adapt and restricted contacts can be compensated, e.g., using online platforms (Long et al., 2021). Accordingly, Stevic et al. (2021) reported that using smartphones to communicate with others was associated with higher friendship satisfaction 1 month later in Austrian adults. As Austrians reported more online than in-person contacts during the initial lockdowns (Nitschke et al., 2021), these alternative ways of contact might be helpful in protecting against loneliness and isolation. Moreover, the crisis might also hold opportunities for strengthened relationships and increased social support. In a German panel study, 55% of adult participants reported having more contact with others during the first lockdown (Schulze et al., 2020), and in a multinational study including 49 nationalities, about half of participants felt more connected to their families and one quarter to their friends since the start of the pandemic (Wu et al., 2021).

Taken together, we conclude that there is an elevated risk for loneliness during the contact restrictions implemented to contain the pandemic but also opportunities for friendships to evolve. There is further need to better understand the factors that influence whether relationships were perceived as support or rather not during the crisis. Additionally, further research is required investigating if and how social support provided by friends is related to mental wellbeing.

In this paper, we focus on different aspects of friendships of Austrian university students during the COVID-19 crisis:

- How did students’ friendships change in the initial and later stages of the pandemic?
- What challenges were students confronted with regarding building and maintaining friendships?
- What role did friendships play for students’ wellbeing during the crisis?

## Research Design and Methods

**Figure 1:**
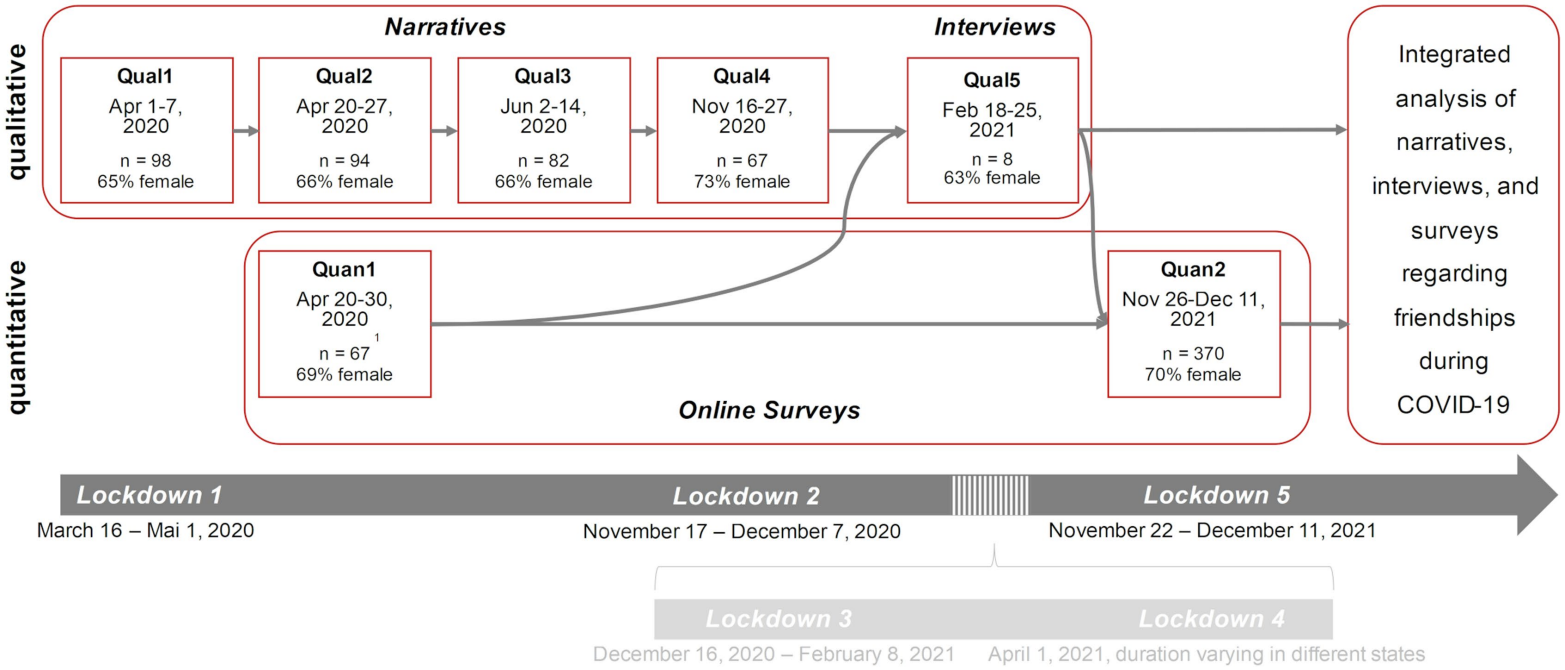
Study design. Qual = data collection for the qualitative study, Quan = data collection for the quantitative study. ^1^repeated measure sample.

This paper is based on a mixed-method approach that comprises a qualitative study with narratives and in-depth interviews and a quantitative study based on the analysis of online survey data (see Figure 1). Both studies investigate the situation of Austrian university students during the COVID-19 pandemic and include longitudinal time frames. The studies were conducted in accordance with the Declaration of Helsinki and were approved by the Board for Ethical Issues of the University of Innsbruck.

The research presented in this article is comprised of multiple stages with each stage building upon the previous and qualitative and quantitative approaches triangularly complementing each other. In the first stage, a comprehensive qualitative study (narratives, Qual1-4, see Figure 1) that investigated University students’ overall socio-spatial conditions during the COVID-19 pandemic was conducted (*cf.* Kaufmann et al., 2020, 2021; Bork-Hüffer et al., 2021). Parallelly a quantitative survey (Quan1) was conducted using an online questionnaire to investigate the situation of university students in Tyrol, Austria. Both studies suggested a further need to investigate changes in university students’ social contacts and friendships during the pandemic and their relevance for wellbeing (Bork-Hüffer et al., 2020; Fischer, 2020; Kulcar et al., 2021). Based on these results, study participants of Qual1-4 were purposefully selected for in-depth interviews that focused on the role of friendships during the pandemic (Qual5). Building upon the preliminary analysis of the narratives and full analysis of the in depth-interviews on friendships (Schneider, 2021), a questionnaire was developed and implemented in another online survey (Quan2). Next, the large longitudinal dataset of narratives was screened for aspects regarding friendships and social contacts and analyzed against the background of the interview and survey results. Finally, results from all data and analysis stages were integrated to answer the research questions.

### The Qualitative Study

The qualitative data used in this article were collected as part of the COV-IDENTITIES project which applied a longitudinal multi-method approach to accompany students through the early stages of the COVID-19 pandemic from April to November 2020. The multi-method design included, among others, written narratives and qualitative online interviews. Figure 1 displays the five qualitative data collection phases (Qual1-5), including sample sizes.

Narratives have proven to be an effective qualitative method for exploring individual experiences with and reflections on complex processes of change (Laughland-Booÿ et al., 2018). The method gives adolescent participants their own voice and room for subjective descriptions and interpretations of their experiences and feelings (see Atkinson, 1998; Pabian and Erreygers, 2019) as well as more time to reflect on, structure, build, and revise their thoughts (see Schulze, 2010). Narratives are particularly suited to conducting research remotely and in crisis times, as they do not rely on stable broadband connections, adhere to research under social distancing conditions, and protect the health of the participants and involved researchers. Follow-up in-depth interviews were conducted with participants, who were purposefully selected from the participants of the narrative writing exercise with the objective to include perspectives from a wide continuum of experiences with friendships during the pandemic.

For the data collection of the narratives, participants received a written storytelling prompt for each narrative exercise in a Microsoft Word file. In the prompt, they were invited to write the narrative, file it in text-processing software, and return the file to the researchers directly after completing it. The 341 collected narratives each range from one to four text pages. There was a larger share of female students among the sample (see Figure 1). Participants’ age varied from 18 to 29 years, while one student was 36 years old. Narratives were submitted by participants in digitized form and thus immediately ready for analysis. Interviews were conducted *via* Zoom. The eight interviews lasted between 18 and 69 min and were transcribed for analysis.

As the narrative writing did not show much change in-between Qual1 and Qual2 since contact restrictions were mostly maintained in that period, in the consecutive presentation of the results, we present data primarily from the collection phases Qual1, Qual3, and Qual4. In addition, qualitative online interviews allowed a review of nearly one year of the pandemic with more specific questions about the topic of friendships of selected study participants. Pseudonyms are used in the presentation of the results.

The software MaxQDA was utilized for conducting a qualitative content analysis of the narratives and interviews following Mayring (2000, 2014).

### The Quantitative Study

The quantitative study started in April 2020 during the first COVID-19 lockdown in Austria (Quan1; Fischer, 2020). German-speaking university students were recruited to participate in an online survey. The data collection was repeated several times during the pandemic. For this paper, we focus on a survey conducted between November 26 and December 11, 2021, (Quan2), when the fifth lockdown was imposed in Austria. At this point, the pandemic had lasted for 20 months and effects on friendships were no longer to be considered preliminary and transient. The survey included detailed scales on students’ friendships based on the qualitative study. Students were recruited *via* a mailing list of the University of Innsbruck and by contacting students who participated in the first survey and deposited their mail addresses. Participants were included in the analysis when living in Austria at the time of the survey and when having a maximum of 5% missing values in the whole survey and no missing values in the relevant scales for this paper.

The final cross-sectional sample consists of *N* = 370 participants. With *n* = 258 (69.7%), the majority was female, *n* = 108 (29.2%) were male, and *n* = 4 (1.1%) did not assign to a binary gender. The mean age was 23.93 years (*SD* = 6.44). To analyze changes in the course of the COVID-19 pandemic, we additionally looked at longitudinal data from students who participated in Quan1 and in Quan2 and could be matched using a code. This code could be optionally entered by participants in each survey and was designed to ensure anonymity. The longitudinal sample included *N* = 67 students, containing of *n* = 46 (68.7%) women and *n* = 20 (29.9%) men with a mean age of 24.52 years (*SD* = 3.51) at Quan2.

#### Measures

The online surveys started by stating information about the research project. Participants could only proceed after providing informed consent. Demographic data were collected in both surveys. The surveys included additional scales that are not presented here as they are not relevant to the research question this article addresses. All presented scales were used in the Quan2 survey.

*Wellbeing* was measured using a German version of the WHO-5 (Bech, 1999; Brähler et al., 2007). The instrument consists of five items that are rated on a six-point Likert response format (0 *never* to 5 *all the time*). Wellbeing was measured in Quan1 and Quan2. Internal consistency for the scale was Cronbach’s *α* = 0.87 in the whole sample in Quan2.

*Pandemic loneliness* was measured using two items (“My social network has become significantly worse due to the crisis.” and “The crisis makes me feel lonely.”). The items were rated on a five-point Likert response format (1 *does not apply at all* to 5 *applies completely*). The items were used with reference to the lockdown instead of the crisis in the Quan1 survey. They were developed as part of an instrument to measure health-promoting behavior (Fischer, 2020). Factor analysis resulted in these two items as one factor (Kulcar et al., 2021). Internal consistency for the scale was Spearman Brown Coefficient = 0.77 in the whole sample in Quan2.

*Contact restrictions during lockdowns* were measured in Quan2 with one item referencing the current situation and one item referencing the first lockdown in retrospective. Participants were asked how much they restricted their physical contact. They rated their number of contacts on a sliding scale with the anchors *no physical contacts at all* and *as many contacts as before the pandemic*.

The following scales were developed based on the results of the qualitative study to specifically address university students’ friendships during the COVID-19 pandemic (Schneider, 2021). The topics of friendship as a resource, challenges for friendships, and changes in friendships emerged from the qualitative analysis and were included in the survey. For all topics, items were phrased based on statements of interview participants. Items and factor analyses are presented in the Supplementary Materials.

*Friendship as a resource* consists of four items (e.g., “My friends are an important support for me during this crisis.”). Answers were rated on a five-point Likert response format (1 *does not apply at all* to 5 *applies completely*). Factor analysis yielded one factor (see Supplementary Table A.1) and internal consistency for the scale was Cronbach’s *α* = 0.83.

*Challenges for friendships* is a seven-item scale (e.g., “It is difficult for me to maintain contact with my friends during this crisis.”) that is answered on a five-point Likert response format (1 *does not apply at all* to 5 *applies completely*). Factor analysis yielded a single factor (see Supplementary Table A.2). Cronbach’s *α* = 0.72 was satisfactory.

*Changes in friendships* consists of nine items that are answered on a five-point Likert response format (1 *does not apply at all* to 5 *applies completely*). Factor analysis resulted in three factors (see Supplementary Table A.3): Loss of friendships (three items, e.g., “I hardly have any contact with some of my friends anymore because of the crisis.,” Cronbach’s *α* = 0.83); Intensification of friendships (four items, e.g., “My friends and I have grown closer through the crisis.,” Cronbach’s *α* = 0.88); Differentiation of friendships (two items, e.g., “The crisis made me realize who is really important to me.,” Spearman-Brown Coefficient = 0.82). These three factors represent changes in friendships also reported in the interviews.

#### Analysis

Quantitative survey data were analyzed using IBM Statistics SPSS, Version 26. To investigate changes between the first lockdown and one and a half years later, the longitudinal sample was analyzed using t-tests for dependent samples based on Quan1 and Quan2. All further analyses are based on Quan2. Mechanisms of predictors of pandemic loneliness were investigated by testing a parallel mediation model with challenges as predictor; loss, intensification, and differentiation of friendships as mediators; and pandemic loneliness as outcome. The buffer hypothesis was tested for the effect of challenges for friendships on wellbeing with friendships as a resource as a moderator. For the mediation and moderation analyses, the macro PROCESS by Hayes (2018) was used. Significance of effects was accessed using 10,000 Bootstrap samples. Effects of friendship variables, including contacts during lockdown, friendships as resource, challenges, and pandemic loneliness, on wellbeing were assessed using a multiple hierarchical regression analysis.

## Results

**Figure 2:**
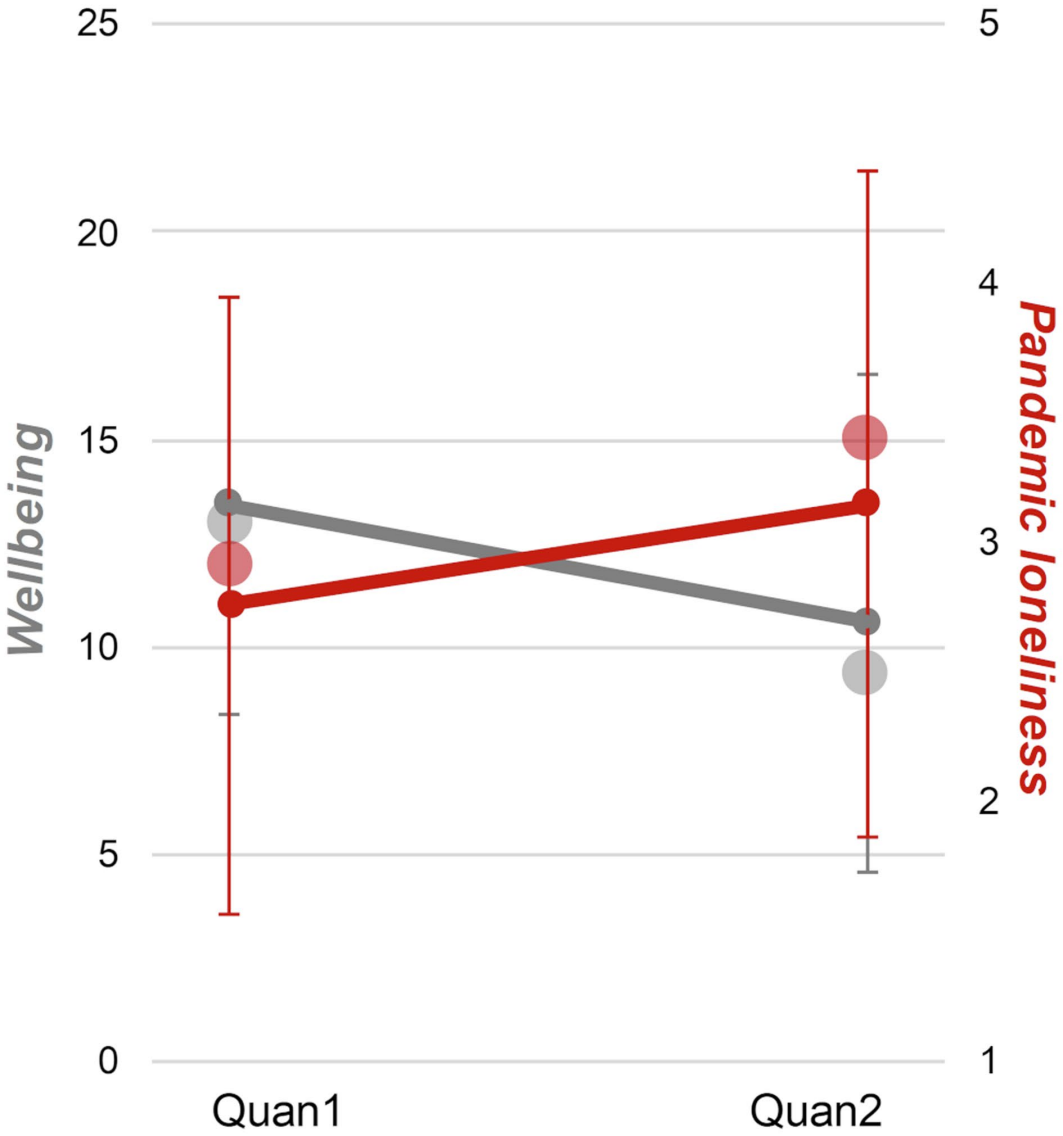
Pandemic loneliness and wellbeing during different lockdowns. Line-diagrams indicate means and standard deviations of the repeated measure sample (*N* = 67), dots indicate means of the whole sample at each lockdown (Quan1 *N* = 334, Quan2 *N* = 370).

The surveys in the beginning of the pandemic (Quan1) and after one and a half years of crisis (Quan2) enabled us to compare students’ perspectives and examine changes. In the 67 students who participated in both surveys, pandemic loneliness increased from *M* = 2.75 (*SD* = 1.19) in April 2020 (Quan1) to *M* = 3.15 (*SD* = 1.29) in November/December 2021 (Quan2). This corresponds to a small but significant effect (*t*(66) = −2.50, *p* = 0.015, *d* = 0.32). Likewise, wellbeing decreased from *M* = 13.40 (*SD* = 4.99) to *M* = 10.57 (*SD* = 6.03) in the repeated measure sample (*t*(66) = 3.83, *p* < 0.001, *d* = 0.51). Means of the whole sample and the repeated-measure sample for both lockdowns are presented in Figure 2. Students who participated in both surveys reported slightly higher wellbeing and lower pandemic loneliness than students who participated only in Quan2 (wellbeing *M* = 9.31, *SD* = 5.24; loneliness *M* = 3.40, *SD* = 1.23). However, this pattern was consistent across surveys and the difference was nonsignificant (wellbeing *t*(89.40) = −1.58, *p* = 0.118; loneliness *t*(368) = 1.48, *p* = 0.140). Wellbeing and pandemic loneliness correlated negatively with *r* = −0.47 during the first lockdown and *r* = −0.45 after one and a half years of the pandemic (both *p* < 0.001).

The effects and developments in the course of the crisis are examined more in detail in the following sections. The qualitative results allow insights into changes and developments from April 2020 (first lockdown, Qual1) over June 2020 (full relaxation of measures, Qual3) to November 2020 (second lockdown, Qual4) and retrospectively as inquired in the qualitative interviews for the first year of the crisis (Qual5). The quantitative results give insight into students’ perspectives and experiences after one and a half years of crisis during the fifth lockdown in Austria (Quan2). Results based on qualitative and quantitative data are merged.

### Challenges for Friendships During the COVID-19 Pandemic

Students experienced multifaceted disruptions to their friendships during the pandemic. Table 1 presents various challenges that were identified in the interviews and narratives and presented to the survey participants in Quan2. The most prominent challenges were the difficulty of making new acquaintances with 68.4% fully agreeing and only a minority of 4.1% not experiencing this problem. The qualitative data (Qual1) of all data collection phases in this regard shows how students were stressed and worried about losing friendships which made them focus on finding ways to maintain existing friendships rather than making new acquaintances. Additionally, opportunities for meeting new people during social activities were lost.

**Table 1:** Percentages of participants experiencing different challenges for their friendships due to the COVID-19 crisis.

	1 (%)	2 (%)	3 (%)	4 (%)	5 (%)
Due to the crisis, it is difficult for me to make new acquaintances	4.1	3.8	7.0	16.8	68.4
To me, online contact is not a good substitute for physical meetings	4.6	10.3	13.0	18.6	53.5
The limited access to important meeting places (e.g., restaurants) puts strains on my friendships	14.3	20.5	21.6	22.4	21.1
During the crisis, it is difficult for me to keep in touch with my friends	15.7	20.8	20.3	28.1	15.1
The mood is less light-hearted when I meet with my friends compared to before the crisis	24.3	18.9	20.3	24.9	11.6
Due to the crisis, I am irritated and I have less patience with my friends	37.8	27.3	16.8	13.8	4.3
Since the COVID-19 pandemic started, I do not know anymore what to talk about with my friends	48.4	23.8	11.1	11.6	5.1

*N = 370. Data collected at Quan2. Response options: 1—does not apply at all to 5—does apply completely*.

Dissatisfaction with online meetings as a substitution for face-to-face meetings with friends represents a further central challenge with 53.5% of participants fully agreeing in Quan2 and only a small share of 4.6% not experiencing difficulties with online meetings at all (see Table 1). Perceived shortcomings of online socializing were reported in the narratives already during the first lockdown (Qual1), when participants characterized online meetings as “not as nice” (Elias, 23, Qual1), “not a proper replacement” (Nele, 23, Qual1), or “simply not a real thing” (Matteo, 21. Qual1) compared to face-to-face meetings. Some thought online meetings were more strenuous and that it was stressful to be constantly available *via* mobile media. Others complained about connectivity issues, not hearing the other party well, the distortion of voices online, or that they could not talk as openly about topics online as in face-to-face meetings. In turn, they particularly missed opportunities for physical closeness and bodily contact – “someone shaking your hand or hugging you” (Juna, 23, Qual1) or “a simple pat on the shoulder” (Alexander, 22, Qual1) –, the possibility of spontaneous social encounters, meeting others “without always having to make an appointment” (Julian, 29, Qual1), or the “input, which [you] get from other people” (Mara, 22, Qual1). As a result, online meetings had been used much less often after the release of the first lockdown (Qual3) and in the following second lockdown (Qual4), confirming the inadequacy perceived in online ways of mingling (see also section “Overall Changes in Friendships During the Crisis in 2020 and 2021”).

Offline activities that were missed, according to the qualitative study, were going out to concerts, to eat out, to a bar, playing, cooking, or doing sports together. The lack of social contacts during everyday activities played a particularly substantial role in Qual1 when students were adhering much stronger to pandemic measures, stay-at-home, and social distancing orders. This was expressed, for example, by Pia (20, Qual1) in early April 2020: “My roommate is not with me while I eat breakfast, my fellow students are not around me while I do my university affairs, and my best friend cannot accompany me to sports. The first impression was that my everyday life has not changed that much, but when you reflect on it, you realize that every action is missing a little something that has a big impact on the big picture.”

A smaller number of students complained about a lack of topics to talk about (Quan2). Particularly during Qual1, participants mentioned that there were no happenings during the lockdown and thus “often nothing new to talk about” (Alexander, 22, Qual1) or that they found it annoying or stressful that “it is anyways only corona that is talked” about (Hannah, 22, Qual1), which led some to avoid meeting with friends (online) during the first lockdown. During the fifth lockdown (Quan2), only 5.1% of participants completely resonated with this challenge. Challenges regarding participants’ mood and associated strains for friendships also showed little prevalence in Quan2. The limited access to meeting places, difficulties to keep in touch with friends, and a weighed down mood were reported by a more substantial proportion of participants (see Table 1).

### Overall Changes in Friendships During the Crisis in 2020 and 2021

As a result of the challenges, friendships changed to varying degrees and in different directions. The results of the qualitative study allow insights into the concrete ways in which the intensity and quality of students’ contacts and friendships were affected. These findings reveal that the ways in which students perceived changes to the intensity and quality of their friendships variegated widely during the first lockdown (Qual1). They reached from evaluations that contacts were reduced “extremely” (Max, 25, Qual1) or “considerably” (Niklas, 22, Qual1) to others who reported spending “most of their days” talking with friends and family online (Jana, 24, Qual1) and thus experienced increased social exchange. Again, others in Qual1 did not feel much change to their friendships and said it was more appropriate to describe their experience as one of “physical distancing” rather than social distancing because “you do not actually give up social contact, just physical closeness” (Alexander, 22, Qual1).

#### Online Contacts as Replacement for In-Person Contacts

In Qual1, differences in the evaluation of changes in friendships were particularly connected to the intensity with which the students and their contacts were able to switch their relationships online and their experience of online socializing as well as their living arrangements. Particularly, WhatsApp, Skype, and telephone, but also Facetime, Facebook, Instagram, Zoom, HouseParty, online games, and online workout platforms were reported as media through which contact was sustained in Qual1. Students usually reported reaching out and experimenting with several of these media for keeping in contact with friends. After the relaxation of the lockdown (Qual3), participants often underlined how their use of social media had “definitely been reduced” (Leonie, 27, Qual3) since face-to-face meetings were possible again. Still, a smaller number of media were used in parallel in the follow-up lockdown of Qual 4 as the appeal of trying out new platforms had diminished. Videotelephony comprised a dominant form of communicating with friends, but also videochat, audio telephony, and text-based messages were used in all data collection phases of the qualitative study (Qual1-5).

Already in Qual1, online socializing was not perceived as an adequate replacement for offline sociability by many participants, but this feeling was expressed even stronger in Qual4, during the second lockdown. However, during the ruptures caused by the stay-at-home orders in Qual1, some found that social media were “a good and important option” (Nora, 23, Qual1) to remain in contact and updated on the well-being of others. Some elaborated on the possibility of social media to connect to those based elsewhere that allowed them to “digitally refresh” (Ben, 24, Qual1) old contacts such as friends from school and their places of origin, friends who had moved elsewhere, or international friends. In contrast, during the second lockdown in November 2020, the narratives (Qual4) reflect that online meetings were much less often used as a replacement for face-to-face meetings. Students complained, for example, that “the ‘pleasure’ in online meetings is gone since everyone anyways needs to participate in compulsory online meetings” (Amelie, 23, Qual4) or that “some people […] got used to not doing so much with different people” (Fiona, 23, Qual4) and thus also decreased their attempts to maintain friendships online.

#### Face-to-Face Meetings During Pandemic Restrictions

Face-to-face meetings with members outside of their own household, which were prohibited during the first lockdown, were still reported by a few students in Qual1. The described meetings took place mostly outside and often only with one, two, or three selected friends, by going for a walk, meeting them in a garden, or “over the fence” (Lena, 25, Qual1). The study participants often underlined in their accounts that this happened while keeping the required distance. Very few reported meeting their friends at their friends’ or their own homes in Qual1. In June 2020 (Qual3), after pandemic measures were loosened, meetings with bigger groups, physical contact, and contacts in indoor spaces became more common again. However, traces of precautions taken to prevent the spread of the virus, prevailed in many of the participants’ stories, reflecting the incision the pandemic had caused to young peoples’ ways of mingling: “The other day we wanted to go out for a drink in the city again for the first time and then we went to sit outside [of the restaurant], even though the weather wasn’t so good, I do not think we would have done that before. So, I spend time with my friends differently than I did before the crisis.” (Louisa, 22, Qual3).

**Figure 3:**
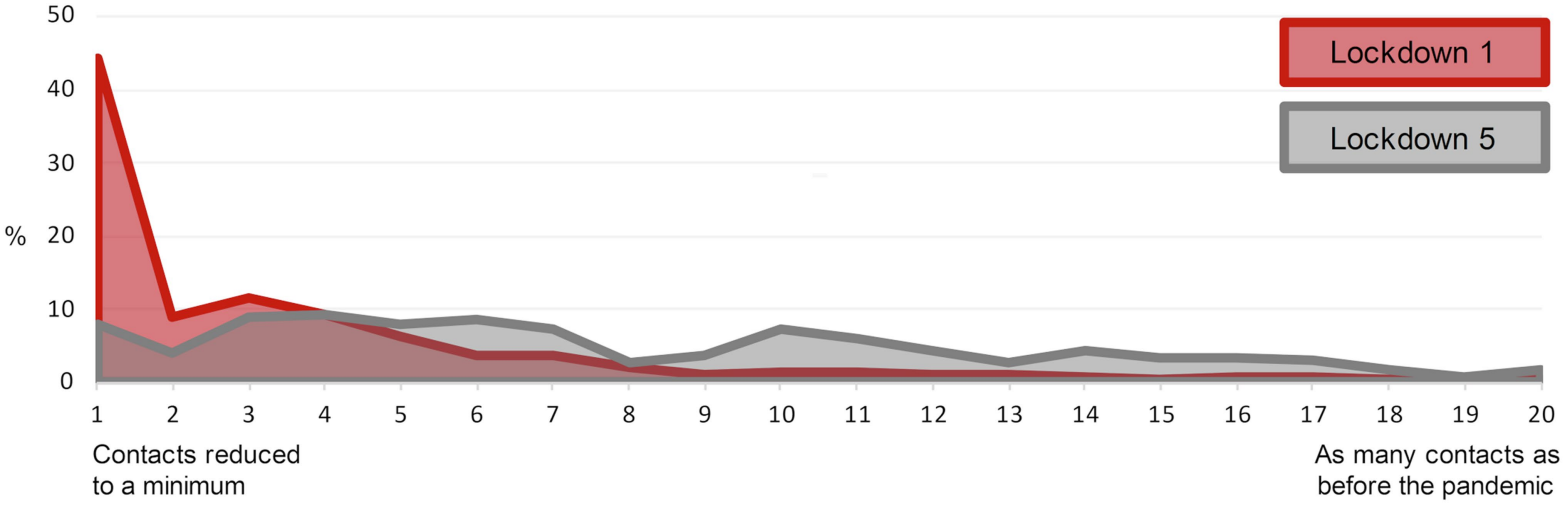
Contacts during different lockdowns based on retrospective evaluation at Quan 2. *N* = 370. Contact restrictions during lockdowns was rated on a 20-point sliding scale.

During the second lockdown in November 2020 (Qual4), students were less strict with reducing face-to-face contacts when compared to the first lockdown (Qual1) and many reported they would meet up with friends more often than during the first lockdown since they perceived it more difficult to “really stick strictly to the restrictions” (Katharina, 23, Qual4). This is illustrated by the quantitative data collected during the fifth lockdown (Quan2). Students were asked to evaluate their restriction of face-to-face contacts currently and retrospectively during the first lockdown. Results are presented in Figure 3. A Wilcoxon test revealed that students perceived a significant and strong decrease in physical contact restriction during the fifth lockdown compared to the first lockdown (*Z* = −13.23, *p* < 0.001, *d* = 1.90).

#### Co-habiting With Friends During Lockdowns

Students’ living arrangements played a considerable role in their experience of socializing and their social resilience, particularly during the first lockdown (Qual1) but for some throughout the pandemic. Some students temporarily moved to live with friends or with their family during the first lockdown, which increased the support they had. Others already shared apartments prior to the pandemic. Although these students also had contact with a limited number of friends during the pandemic overall, they reported that spending time with their roommates helped them mitigate isolation and many benefitted from the mutual support: “I must mention that I do not live alone but with my favorite person, which of course makes the curfew more pleasant. We finally have time to cook together, build puzzles, do crossword puzzles, play games, or watch movies. I enjoy that a lot.” (Amelie, 23, Qual1).

Then again, for some, this advantage was short-lived when “in an exceptional situation […] being now together 24/7” (Anna, 23, Qual1), sometimes in cramped living conditions, shared living arrangements caused stress and conflict among students. The relaxation in summer 2020 brought relief for some of them. For a few, the mental strains of co-living during the pandemic resulted in them moving out of shared living arrangements. To mitigate changes to online and offline sociability during the pandemic, students reported developing new rules in Qual1. These encompassed, for example, stricter rules on duties that roommates in shared apartments would hold but also rules that specified what could (not) be talked about in online meetings: “We are very strict with the rules and also sit down together over and over again to discuss if we are still at a dead end. We are open and honest with each other to make it work, but also strict with each other, which has led to many moral discussions. Everyone sets their boundaries differently, which is why it’s hard to “correct” others.” (Helena, 21, Qual1). “But since we all noticed that you should also have a space where current events should not be a topic for at least 2 h, just to get some distance for once, we decided not to bring up the COVID-19 pandemic during our conferences. It’s working really well now, and it’s hugely important for oneself.” (Elisa, 24, Qual1).

#### Loss, Intensification, and Differentiation of Friendships

All in all, in the qualitative data collection rounds Qual3-5 throughout 2020, reports of overall reduced social contacts became more prevalent. Online socializing was by then no longer considered an adequate replacement by most, and a persistent decline in the number of relationships the longer the pandemic lasted, can be witnessed: “Yes, I just think that the worlds have shrunk a bit and so has the contact between them. And to be honest, I also think that some friendships have really suffered drastically as a result.” (Helena, 21, Qual5).

This also resulted in profound changes in friendships that were particularly described in Qual3-5. On one hand, there were reports of the intensification of specific friendships—particularly with friends to whom face-to-face contact was maintained throughout the pandemic. On the other hand, as Julian (29, Qual3) noted, “particularly loose friendships fall into oblivion” the longer the pandemic lasted. Overall, the young adults described how they reflected more upon the quality of their existing friendships and more reflexively thought about which friendships to maintain in the face of the changed circumstances. They reported, for example, to focus on those who “were the favorite [people] to have around” (Emil, 25, Qual3), “who [they] really care about” (Alina, 26, Qual3), and who had shown care for them during the difficult time of the pandemic. Furthermore, in various phases of the qualitative study, participants reported a renewed appreciation of friendships and social contacts overall in their lives: “However, I already know that after the pandemic hopefully improves in a timely manner, I will pay much more attention to my social contacts as I have sensed how important they are for overall satisfaction and for ‘soul life’. Whereas a few months ago I might have said that meeting up would not work out due to stress, current experiences have made me push meeting up ahead of work.” (Theo, 28, Qual1).

**Figure 4:**
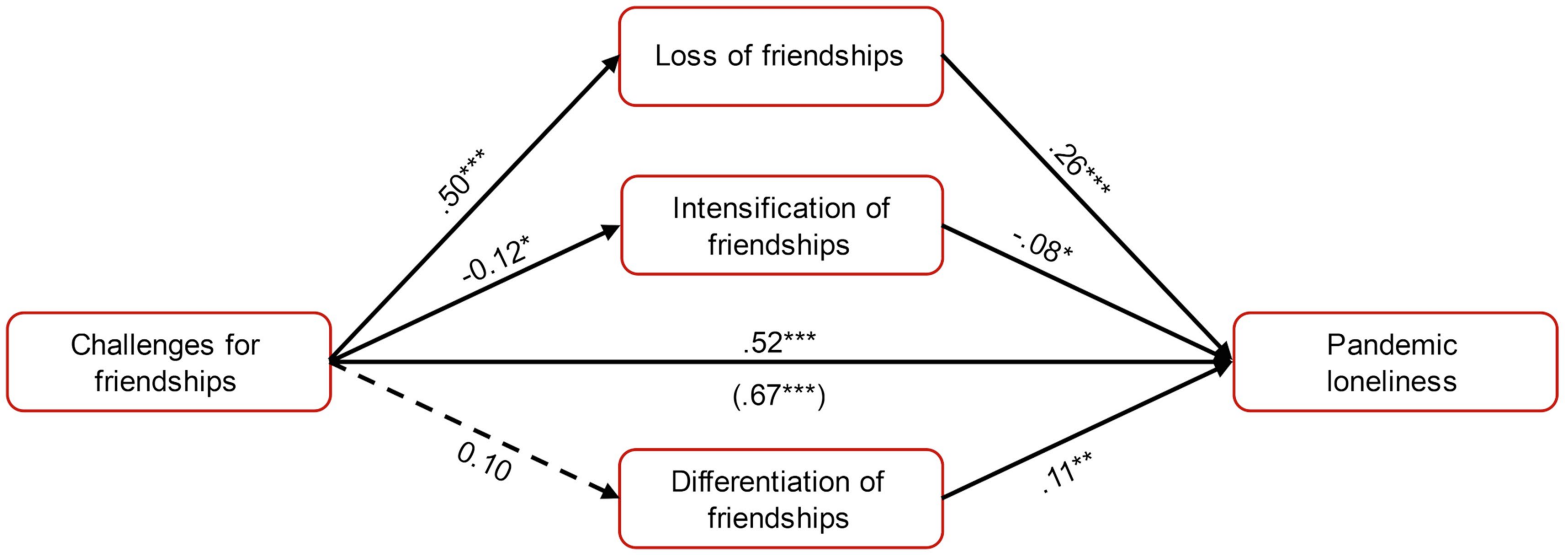
Mediation model for challenges for and changes in friendships. Dotted lines represent non-significant effects. ***p < 0.001, **p < 0.01, *p < 0.05.

The quantitative data (Quan2) were employed to assess how these changes in friendships relate to challenges posed by the pandemic and pandemic loneliness. Challenges for friendships had significant effects on loss (*B* = 0.83, *SE* = 0.08, β = 0.50, *p* < 0.001) and intensification of friendships (*B* = −0.16, *SE* = 0.07, *β* = −0.12, *p* = 0.025). Challenges were associated with increased loss of friendships and decreased intensification. There were no effects on differentiation of friendships (*B* = 0.17, *SE* = 0.09, *β* = 0.10, *p* = 0.059). The effect of challenges on pandemic loneliness (*B* = 1.09, *SE* = 0.06, *β* = 0.67, *p* < 0.001; total effect) decreased when the changes in friendships variables were included into the model; however, a direct effect was maintained (*B* = 0.85, *SE* = 0.07, *β* = 0.52, *p* < 0.001; direct effect). Simultaneously, loss of friendships was associated with increased pandemic loneliness (*B* = 0.25, *SE* = 0.04, *β* = 0.26, *p* < 0.001) as was differentiation of friendships (*B* = 0.11, *SE* = 0.04, β = 0.11, *p* = 0.008) while intensification was associated with decreased pandemic loneliness (*B* = −0.10, *SE* = 0.05, *β* = −0.08, *p* = 0.044). However, challenges exerted an indirect effect on pandemic loneliness only mediated by loss of friendships (*B* = 0.21, *SE* = 0.04, 95% CI [0.16, 0.34]) but there was no indirect effect mediated by intensification (*B* = 0.02, *SE* = 0.01, 95% CI [−0.01, 0.05]) or by differentiation of friendships (*B* = 0.02, *SE* = 0.01, 95% CI [−0.01, 0.05]). The effects are depicted in Figure 4.

### Friendships as a Resource During the COVID-19 Pandemic and Students’ Wellbeing

In Table 2, correlations between the friendship scales, wellbeing, and demographic data (age, gender) are presented. Wellbeing was higher in students who restricted their face-to-face contact less, who utilized friendships more as a resource, who perceived fewer challenges for friendships, and who felt less isolated. Challenges for friendships correlated strongly with pandemic loneliness and students who used their friendships as a resource during the crisis reported both fewer challenges and felt less isolated. Gender was not associated with wellbeing, but women used their friendships more as resources while men experienced more challenges for their friendships. Older students reported both higher wellbeing and less pandemic loneliness.

**Table 2:** Correlations of friendship variables, wellbeing and demographics at Quan2.

		1	2	3	4	5	6
1	Gender						
2	Age	0.18^**^					
3	Wellbeing	−0.01	0.22^***^				
4	Contact restrictions	0.04	−0.00	0.11^*^			
5	Friendship as a resource	−0.14^**^	−0.05	0.22^***^	0.17^**^		
6	Challenges for friendships	0.15^**^	−0.09	−0.41^***^	−0.07	−0.33^***^	
7	Pandemic loneliness	0.08	−0.22^***^	−0.45^***^	−0.15^**^	−0.39^***^	0.67^***^

*N = 370, data collection Quan2; Gender 0 = female, 1 = male and others.*

* *p < 0.05*;

** *p < 0.01*;

*** *p < 0.001*.

#### Appreciation of Friends’ Social Support

In the qualitative data, participants described how their friends aided them in coping with the crisis. They explained how they purposefully decided to have in-person meetings because they “will all go crazy if [they] do not” (Valentina, 24, Qual1), that they did not feel lonely thanks to living with friends, and how talking with friends helped them to make their “fears clear to [them] and to play them out a bit, so that [they] could then put them aside again” (Finja, 26, Qual1) during the early stages of the crisis in Qual1. Students explained how they realized how much their friends meant to them and how important they were for their emotional wellbeing. As restrictions loosened, participants explained how they looked forward to seeing their “favorite persons again and spending time with them (Nora, 23, Qual3) and how they met their friends “way more consciously and really enjoy it” (Maja, 22, Qual3). Anna (23, Qual3) described the importance of meeting friends in person again: “I remember the first meeting with my group of friends very well. Here, I realized how important contact with other people is and I think no one has ever looked forward to a meeting as much as this one.”

This importance of friends stayed prevalent during the second lockdown as participants explained that they met specific friends because it was “very important for [their] psychological wellbeing” (Katharina, 23, Qual4). One participant reviewed in an interview how they deliberately chose to violate the measures and meet friends because it was essential for staying mentally healthy: “And that’s just when you do something like tobogganing or climbing again, then you realize how much that actually helps you or how well you feel after you have really met a few people again. So certainly the direct contact with friends I think helps the most for me.” (Alexander, 22, Qual5).

Some students explicitly stated how important it was to be in contact with their friends when they were not feeling well: “You also realized that somehow, I do not know, everything can go badly, but as long as you have each other and get through it together, even if everyone feels bad you at least feel bad with the people you love.” (Sophia, 22, Qual5).

#### Shared Experiences With Friends

Besides the general importance of connecting with friends in person, students explained during the in-depth interviews how being able to share difficult situations with their friends helped them. Thereby it was important to them that friends were in similar situations because students felt they otherwise might not be able to truly understand them. For example, they felt like they needed other university students to share experiences about problems at university due to the crisis because friends who were not students “cannot really empathize with a student” (Alexander, 22, Qual5). Therefore, they expected the most support from people who are in a similar situation as themselves. The shared experiences enabled a mutual understanding without having to explain much: “Most of it was actually common whining, I would say. You somehow coax each other but I feel like it was most beneficial when the other person was just as upset as oneself. Shared sorrow is half the sorrow somehow. I think it was extremely supportive when you realized the others are feeling as I do.” (Juna, 23, Qual5).

The shared experiences were a foundation for providing “permanent support” (Sophia, 22, Qual5) for each other instead of specific moments of support.

#### Instrumental Support by Friends and Peers

In addition, participants reported receiving different forms of instrumental support. This support was predominantly focused on the university and was relevant at different stages of the pandemic. Students reported “completing tasks for university together” (Milan, 25, Qual1) with friends and roommates and creating online study groups in Qual1. As restrictions loosened during Qual3 but access to libraries and the university was still restricted, study groups stayed an important support system in students’ everyday lives with groups now meeting in person more frequently, for example by “repurposing unused common rooms in [their] residential buildings” (Alexander, 22, Qual3). By meeting regularly for university tasks and studying, students were able to support each other and gain structure and regularity. They were able to “motivate each other but also relax together” (Sophia, 22, Qual4).

#### Effects of Friendships on Wellbeing

The quantitative data at Quan2 were used to analyze the effects of restricted contacts, utilization of friendships as resources, challenges for friendships, and pandemic loneliness on wellbeing after one and a half years of crisis in a regression analysis. As gender and age significantly correlated with some of the variables, both were used as control variables and entered into the model first. Subsequently, a hierarchical approach was implemented. As presented in Table 3, how much students reduced their contacts significantly predicted wellbeing with participants who had a similar number of contacts as before the pandemic reporting higher wellbeing (*B* = 0.12, *SE* = 0.05, *β* = 0.11, *p* = 0.031). However, this effect was rendered nonsignificant once other variables were entered into the model. The same was true for the utilization of friendships as a resource during the crisis. Students who were able to use their friendships as a resource reported higher wellbeing (*B* = 1.26, *SE* = 0.30, β = 0.22, *p* < 0.001) but this effect was nonsignificant after challenges for friendships were entered into the model. Students who reported more challenges for their friendships had lower wellbeing (*B* = −2.57, *SE* = 0.35, β = −0.36, *p* < 0.001). The effect was smaller but still significant after pandemic loneliness was entered as final predictor into the model (*B* = −1.56, *SE* = 0.44, β = −0.22, *p* < 0.001). Students who felt more lonely due to the crisis reported lower wellbeing (*B* = −1.05, *SE* = 0.28, β = −0.24, *p* < 0.001). The final model explained 25% of variance in wellbeing (*F*(6,363) = 19.92, *p* < 0.001, *R^2^* = 0.25). For cross-validation, the coefficients were used to predict wellbeing in a sample of students not living in Austria during the data collection (*N* = 94; *n* = 55 Germany, *n* = 26 Italy, *n* = 13 other). In this sample, *R^2^* = 0.29 was achieved which indicates that the results are robust across countries even if these countries are currently not under lockdown such as Germany. Finally, we tested the buffer hypothesis of social support by using friendships as a resource as a moderator for the effect of challenges on wellbeing. There was no significant interaction effect (*B* = −0.10, *SE* = 0.38, *p* = 0.788), thus the buffer effect was not confirmed.

**Table 3:** Regression analysis to predict wellbeing.

	Model 1		Model 2		Model 3		Model 4		Model 5	
	*β*	Δ*R*^2^	*β*	Δ*R*^2^	*β*	Δ*R*^2^	*β*	Δ*R*^2^	*β*	Δ*R*^2^
Gender	−0.05	0.05^***^	−0.05	0.01^*^	−0.02	0.05^***^	0.03	0.11^***^	0.03	0.03^***^
Age	0.23^***^		0.23^***^		0.23^***^		0.19^***^		0.14^**^	
Contacts			0.11^*^		0.07		0.06		0.04	
Friendship as a resource					0.22^***^		0.10		0.06	
Challenges for friendships							−0.36^***^		−0.22^**^	
Pandemic loneliness									−0.24^***^	
*R^2^*	0.05		0.06		0.11		0.22		0.25	
*F*	9.62^***^		8.03^***^		10.86^***^		20.43^***^		19.92^***^	

*N = 370, data collection Quan2. Gender 0 = female, 1 = male and others.*

* *p < 0.05*;

** *p < 0.01*;

*** *p < 0.001*.

#### Advantages of the Crisis

However, some participants were also able to discover advantages in the restrictions. This mainly related to enjoying meetings with smaller, more intimate groups of people compared to larger groups: “Friends [...] This is where it shifted from ‘a lot of time with a lot of different people’ to ‘a lot of time with a smaller group of people’. This, I notice, relaxes me a lot” (Adrian, 21, Qual1).

Especially in Qual1, students explained how the restrictions helped them relax as they “never worried of missing out on something” (Amelie, 23, Qual1) and they “enjoy time alone without social obligations” (Sophie, 23, Qual1). During Qual3, some students started worrying about enjoying the time alone too much wondering if they were “antisocial” (Aaron, 26, Qual3) or that “switching to a ‘normal’ life afterward will be overwhelming” (Malia, 23, Qual4). Others “continue[d] to enjoy the time alone” (Emily, 23, Qual4) during the second lockdown at Qual4.

## Discussion

Overall, the results of both the qualitative and the quantitative study indicate a substantial impact of the pandemic and the associated measures on university students’ friendships. These impacts were based on different challenges students faced during the pandemic and caused changes in the way participants interacted with friends but also in their friendship network in general. This included both the loss of friends and the intensification of friendships. Simultaneously, friendships and frequent contact with friends were important for students’ mental wellbeing. Friends supported each other in coping with the crisis and students deployed different strategies to keep up a supportive network of peers. However, social support provided by friends was not effective in protecting against the negative impacts of the pandemic caused by disruptions of social contacts.

### How Did Students’ Friendships Change in the Initial and Later Stages of the Pandemic?

The pandemic had a persistent negative effect on both building and maintaining friendships. Participants reported increased pandemic loneliness in the course of the crisis despite restricting their face-to-face contacts less. Participants having more in-person contacts at later stages of the pandemic might be a result of this increasing loneliness. Students were less willing to restrict their contacts as the pandemic prolonged and they suffered increasingly under the measures while they did not know how much longer they would have to refrain from meeting their friends. While they were largely prepared to give up on in-person meetings at the beginning of the pandemic and online meetings were deemed an acceptable replacement for a short time, they quickly noticed the strain these contact restrictions put on their mental health they were not willing to accept. As a result, many students resumed meeting their friends in person, but our results indicate that the pandemic still was a burden for friendships. This resulted in the loss of friendships for some. This loss often concerned loose friendships while close friends were maintained in many cases. However, maintaining friendships is often costly regarding both time and effort (Dunbar, 2018) and friendships can be lost when contact and joint activities are too rare (Roberts and Dunbar, 2011), so this loss was not restricted to loose friendships. Contrarily, there were also reports of intensified and strengthened friendships which is in line with other studies on students (Vaterlaus et al., 2021). Particularly friends who were met face-to-face and who were experienced as being supportive and trustworthy did not decline in their importance and contact was maintained.

Even though strengthened relationships represent one result of the pandemic, the changing pattern of social networks might nonetheless have long-term implications. Young adulthood is usually characterized by a large network of acquaintances and friends compared to other age groups (Wrzus et al., 2013) and this network is crucial for their future lives (Roisman et al., 2004). As people get older, the size of their social networks typically decreases (Wrzus et al., 2013). During the pandemic and particularly during periods with strict contact restrictions such as lockdowns, young adults are not able to build a large circle of friends and acquaintances. It is unknown if this will be compensated by making more new contacts after the pandemic ends or by a smaller decrease of social networks as the young adults age especially due to the intensification of friendships in some. It is possible that some people will not be able to compensate for the missing opportunities of connecting with others during the formative period of young adulthood and will therefore be missing social support later in their life. At this point, we can only hypothesize about such future effects.

### What Challenges Were Students Confronted With Regarding Building and Maintaining Friendships?

The changes in friendships can be led back to the challenges for friendships posed by the pandemic and the associated restrictions. During the first lockdown, participants strongly restricted their face-to-face contacts. However, many perceived this mainly as physical distancing while successfully shifting socializing online. The degrees of this shift to online communication varied and so did experiences and satisfaction. As Juvonen et al. (2022) found in young adults in California, satisfaction with digital communication was predictive of their socio-emotional wellbeing during this pandemic. But for most participants in our study, online contacts were not deemed an appropriate replacement for in-person meetings as the pandemic prolonged. Important meeting places and offline activities were lost and physical contact and closeness as well as the opportunities to meet new people were dearly missed. This is in line with Andresen et al. (2020) who concluded that digital contacts are helpful for organizing friendships but not for maintaining them. Then again, the ruptures to social relationships that the pandemic caused might as well have been considerably larger without the possibility to keep at least a certain level of connection to others online.

Regarding the effects of challenges on changes in friendships, perceiving more challenges was associated with less intensification and more loss of friendships. While the effect on the intensification of friendships was weak, there was a strong effect of challenges on the loss of friends. Additionally, challenges were directly associated with more pandemic loneliness. These results suggest that not only the loss of friends was relevant but also the loss of a lifestyle characterized by regular in-person meetings, going out in public places, and making new acquaintances. The COVID-19 measures not only made the maintenance of friendships difficult but also impaired the typical student lifestyle which left students feeling restricted and lonely. Intensification of friendships was not able to meaningfully protect against this effect. There also might be reverse effects with people who were able to bolster their friendships interpreting the challenges as less prevalent while students who lost friends experienced them as prevailing. However, considering the predominant effect of challenges on pandemic loneliness, the implications remain largely unchanged with not only changes in friendships but also restrictions in student lifestyle contributing to loneliness and isolation.

### What Role Did Friendships Play for Students’ Wellbeing During the Crisis?

In the qualitative data, students explained how important their friends were to them during the crisis and how they supported them in maintaining their mental health. Contrarily, in the quantitative analyses, utilization of friendships as a resource did not have significant effects on wellbeing beyond the effects of challenges for friendships. Challenges for friendships seem to impair wellbeing regardless of available support from friends. The buffer hypothesis was not supported either. At the same time, experiencing challenges for friendships and feeling lonely was associated with reduced wellbeing. The results indicate that, during this pandemic, being in contact with friends represents a fundamental need for university students and failure to meet this need results in impaired wellbeing (Baumeister and Leary, 1995).

The failure to find significant effects of utilization of friendships as a resource can have several reasons. First, the impairment of friendships might prevent them from serving as a resource themselves. Students might be too preoccupied with maintaining their friendships to effectively employ them as a resource that protects them against pandemic stressors. This can be interpreted with regards to the Conservation of Resources Theory (Hobfoll, 1989) that posits the possibility of loss spirals. Loss spirals are characterized by the loss of one resource triggering the loss of further resources in accelerating speed as people are no longer able to employ their resources to protect against resource loss. The affected person can merely try to limit the damage. This might be the case for students whose friendships—and maybe also other parts of their lives—are severely impacted.

Second, many young adults move away from their hometowns to attend university and must leave their circle of friends behind which leaves them lonely even without a pandemic around (Juvonen et al., 2022). Therefore, they cannot rely on their former support system, but at the same time, the crisis makes the acquisition of new friends difficult. Hence, it could be argued that friendships were overall a limited social resource for university students during the pandemic.

Third, relationships often suffer if one person is in need of support, but close ones do not respond to this need because they are lacking the capacity to support others or because they do not realize the person is in need (Thoits, 2011). Since the COVID-19 crisis is challenging for most people, providing friends with support might be impeded and even if someone has the capacity to provide support, the person needs to become aware of the need for support. Realizing that friends are struggling might be more difficult with meetings restricted to online settings. The results of the qualitative study also reflect how online platforms were not perceived as a place where students felt comfortable to share their worries and not adequate for providing care to others. People might indicate that support provided by their friends is appropriate because they realize that it is the best they can do under the given circumstances. However, this support might not be sufficient to buffer the effects of pandemic strains.

Another aspect to consider is the assumption that people who have suffered through the same crisis as the person in distress can supply them with specific support (Thoits, 2011). Everyone being in a similar situation during the pandemic might indeed be comforting and students themselves explained that shared experiences are important to them. However, as none of the young adults got through a pandemic so far, providing support and hope based on experience might be more difficult compared to other situations. Results of the qualitative study also showed that being together in the same situation and being faced with restrictions to mutual offline activities also meant less distraction offered by others.

### Strengths and Limitations

The present study employed a mixed-method approach and covers a broad period of time during the pandemic. The combination of qualitative approaches for in-depth analysis of students’ experiences with quantitative approaches for the investigation of statistical effects in a larger sample allows well-founded conclusions regarding the friendships of university students. Nonetheless, our conclusions only regard university students in Tyrol, Austria, and cannot be generalized to other samples. This especially applies during pandemic times as every country and every region—sometimes even every university—employed different measures and restrictions. The utilization of measures that were specifically designed for this population further prevents generalizability. However, cross-validation with a sample of university students living in other countries with different measures during the pandemic provided promising results.

### Conclusion and Implications

Taken together, the COVID-19 crisis put profound strains on university students’ friendships. Our results indicate considerable impacts that sustain one and a half years into the pandemic and made the utilization of friendships to support resilience difficult. The changes in friendships and friendship networks might have long-term implications for current young adults. Our results suggest a need for further research specifically on friendships during the COVID-19 crisis. Research should not only focus on analyzing effects retrospectively but should also investigate lasting effects and incorporate efforts to assist young adults in overcoming this crisis. This includes, among others, research on the effectiveness of different interventions. This is crucial as loneliness can be associated with maladaptive reactions such as social withdrawal (Vasileiou et al., 2019) or cognitive biases (Hawkley and Cacioppo, 2010) that might require specific interventions in some cases. Short-term interventions after the pandemic should also be considered to provide young adults the opportunity to compensate for the lack of contacts during the pandemic. These can include peer and buddy systems at universities or events. If further contact restrictions are necessary, measures should be implemented in a way that allows maintenance of contacts, for example in contact clusters (Wu et al., 2021).

## Data Availability Statement

The quantiative data presented in this article are included in the Supplementary Material. The qualitative data are not openly available to protect the participants’ privacy. Further inquiries can be directed to the corresponding author.

## Ethics Statement

The studies involving human participants were reviewed and approved by the Board for Ethical Issues, University of Innsbruck. The patients/participants provided their written informed consent to participate in this study.

## Author Contributions

TB-H and A-MS contributed to the conception and design of the qualitative study, collected data for the qualitative study, and performed the qualitative analysis. VK contributed to the conception and design of the quantitative study, collected data for the quantitative study, performed the statistical analysis, and wrote the first draft of the manuscript. VK and TB-H wrote sections of the manuscript. All authors contributed to the article and approved the submitted version.

## Funding

This work was supported by funding from Förderkreis 1669 of the University of Innsbruck (grant number 329327; quantitative study). The sponsors were not involved in the study design; collection, analysis, or interpretation of data; writing of the article; or the decision to submit the article for publication. The Open Access Publication of this article was supported by the Open Access Fund of the University of Innsbruck.

## Conflict of Interest

The authors declare that the research was conducted in the absence of any commercial or financial relationships that could be construed as a potential conflict of interest.

## Publisher’s Note

All claims expressed in this article are solely those of the authors and do not necessarily represent those of their affiliated organizations, or those of the publisher, the editors and the reviewers. Any product that may be evaluated in this article, or claim that may be made by its manufacturer, is not guaranteed or endorsed by the publisher.

## Supplementary Material

The Supplementary Material for this article can be found online at: https://www.frontiersin.org/articles/10.3389/fpsyg.2022.880646/full#supplementary-material
