# Supplementary material for: Getting Through the Crisis Together: Do Friendships Contribute to University Students’ Resilience During the COVID-19 Pandemic?
Source: Front Psychol. 2022 May 16;13:880646. doi: 10.3389/fpsyg.2022.880646 (PMC9149295; doi:10.3389/fpsyg.2022.880646)
Supplement: Supplementary file 2 [file Data_Sheet_1.docx]

Supplementary Material

# Factor analyses of newly developed scales

Note that English versions of the items were not language checked or backtranslated.

**Table A.1.** Factor analysis for friendships as a resource providing a one-factor solution.

|  | *M* | *SD* | Factor loading |
| --- | --- | --- | --- |
| My friends give me the feeling that we will get through the crisis together.  [Meine Freund:innen geben mir das Gefühl, dass wir die Krise gemeinsam durchstehen.] | 3.54 | 1.14 | .89 |
| My friends and I support each other during this crisis.  [Meine Freund:innen und ich unterstützen uns während dieser Krise gegenseitig.] | 3.75 | 1.09 | .88 |
| My friends are an important support for me during this crisis.  [Meine Freund:innen sind für mich eine wichtige Stütze während dieser Krise.] | 3.89 | 1.04 | .78 |
| I would have liked more support from my friends during this crisis. (R)  [Ich hätte mir während dieser Krise mehr Unterstützung von meinen Freund:innen gewünscht.] | 3.69 | 1.28 | .48 |
| Cronbach’s α |  |  | .83 |
| *M* |  |  | 3.72 |
| *SD* |  |  | 0.93 |

*Note. N* = 370. Factor matrix of a main axis factor analysis with extraction of Eigenvalues > 1 and oblique (direct oblimin) rotation with Kaiser Normalization. Reverse-scored items are coded with (R). Kaiser-Meyer-Olkin measure = .78, χ^2^(6) = 722.65, *p* < .001. One item („When I am with my friends, I can forget about the crisis.”) was removed from the scale due to reductions in the internal consistency and low factor loading of .30.

**Table A.2.** Factor analysis for challenges for friendships providing a one-factor solution.

|  | *M* | *SD* | Factor loading |
| --- | --- | --- | --- |
| The limited access to important meeting places (e.g., restaurants) puts strains on my friendships.  [Der beschränkte Zugang zu wichtigen Treffpunkten (z.B. Gastronomie) belastet meine Freundschaften.] | 3.15 | 1.35 | .64 |
| During the crisis, it is difficult for me to keep in touch with my friends.  [Es fällt mir schwer, während dieser Krise den Kontakt zu meinen Freund:innen aufrecht zu erhalten.] | 3.06 | 1.31 | .57 |
| Since the COVID-19 pandemic started, I do not know anymore what to talk about with my friends.  [Seit der COVID-19 Pandemie weiß ich nicht mehr, worüber ich mit meinen Freund:innen sprechen soll.] | 2.01 | 1.24 | .55 |
| Due to the crisis, it is difficult for me to make new acquaintances.  [Durch die Krise fällt es mir schwer, neue Bekanntschaften zu schließen.] | 4.42 | 1.05 | .51 |
| Due to the crisis, I am irritated and I have less patience with my friends.  [Durch die Krise bin ich genervt und habe weniger Geduld mit meinen Freund:innen.] | 2.19 | 1.21 | .50 |
| The mood is less light-hearted when I meet with my friends compared to before the crisis.  [Die Stimmung bei Treffen mit meinen Freund:innen ist weniger unbeschwert als vor der Krise.] | 2.81 | 1.36 | .50 |
| To me, online contact is not a good substitute for physical meetings.  [Online-Kontakte sind für mich kein guter Ersatz für physische Treffen.] | 4.06 | 1.22 | .35 |
| Cronbach’s α |  |  | .72 |
| *M* |  |  | 3.10 |
| *SD* |  |  | 0.76 |

*Note. N* = 370. Factor matrix of a main axis factor analysis with extraction of Eigenvalues > 1 and oblique (direct oblimin) rotation with Kaiser Normalization. Kaiser-Meyer-Olkin measure = .79, χ^2^(21) = 401.61, *p* < .001.

**Table A.3.** Factor analysis for changes in friendships providing a tree-factor solution.

|  | *M* | *SD* | Intensification | Loss | Differentiation |
| --- | --- | --- | --- | --- | --- |
| My friendships have become even more intense as a result of the crisis.  [Meine Freundschaften sind durch die Krise noch intensiver geworden.] | 2.41 | 1.22 | **.98** | -.01 | .06 |
| My friends and I have grown even closer through the crisis.  [Meine Freund:innen und ich sind durch die Krise noch enger zusammen gewachsen.] | 2.61 | 1.23 | **.92** | -.01 | .02 |
| During the crisis, my friendships were strengthened.  [Während der Krise wurden meine Freundschaften gestärkt.] | 2.41 | 1.22 | **.85** | -.10 | -.03 |
| Since the crisis, I have fewer but closer contacts.  [Seit der Krise habe ich weniger, aber engere Kontakte.] | 2.91 | 1.32 | **.45** | .16 | -.22 |
| Through the crisis I have lost friends.  [Durch die Krise habe ich Freund:innen verloren.] | 2.61 | 1.57 | .09 | **.94** | .07 |
| Some of my friends broke off contact during the crisis.  [Manche meiner Freund:innen haben während der Krise den Kontakt abgebrochen.] | 2.18 | 1.44 | .00 | **.78** | .02 |
| I hardly have any contact with some of my friends anymore because of the crisis.  [Zu manchen meiner Freund:innen habe ich durch die Krise kaum noch Kontakt.] | 3.44 | 1.42 | -.11 | **.66** | -.05 |
| The crisis made me realize who is really important to me.  [Durch die Krise habe ich gemerkt, wer mir wirklich wichtig ist.] | 3.29 | 1.36 | -.03 | -.03 | **-.94** |
| The crisis made me realize who I can rely on.  [Durch die Krise habe ich gemerkt, auf wen ich mich verlassen kann.] | 3.35 | 1.43 | .05 | .00 | **-.74** |
| Cronbach’s α |  |  | .88 | .83 | .82 ^a^ |
| *M* |  |  | 2.58 | 2.74 | 3.32 |
| *SD* |  |  | 1.07 | 1.27 | 1.29 |

Note. *N* = 370. Pattern matrix of a main axis factor analysis with extraction of Eigenvalues > 1 and oblique (direct oblimin) rotation with Kaiser Normalization. Factor assignment is bold. Kaiser-Meyer-Olkin measure = .77, χ^2^(36) = 1931.93, *p* < .001. ^a^Spearman-Brown coefficient.
